# Ipsilateral transient amaurosis, mydriasis and light reflex absence after subconjunctival local anesthesia with mepivacaine in three patients with refractory glaucoma – a case report

**DOI:** 10.1186/s12886-019-1202-2

**Published:** 2019-08-28

**Authors:** Katharina Knoll, Kristine Chobanyan-Jürgens, Dirk O. Stichtenoth, Ingo Roland Volkmann, Katerina Hufendiek, Carsten Framme

**Affiliations:** 1 Department of Ophthalmology, University Eye Hospital, Hannover Medical School, Carl-Neuberg-Str.1, 30625 Hannover, Germany; 2 Institute of Clinical Pharmacology, Hannover Medical School, Carl-Neuberg-Str.1, 30625 Hannover, Germany

**Keywords:** Glaucoma, Mepivacaine, Cyclophotocoagulation, Amaurosis, Mydriasis

## Abstract

**Background:**

The subconjunctival anesthesia with local anesthetics is considered as a low-risk procedure allowing ocular surgery without serious complications typical for retro- or parabulbar anesthesia, especially in patients with preexisting Optic Nerve damage. We report development of ipsilateral transient amaurosis accompanied with mydriasis and both, direct and consensual light response absence.

**Case presentation:**

Three patients with advanced refractory glaucoma undergoing laser cyclophotocoagulation (CPC) for intraocular pressure lowering experienced these adverse effects just few minutes after subconjunctival injection of mepivacaine 2% solution (Scandicaine® 2%, without vasoconstrictor supplementation).

The vision was completely recovered to usual values in up to 20 h after mepivacaine application. Extensive ophthalmological examination, including cranial magnetic resonance imaging (MRI), revealed no further ocular abnormalities, especially no vascular constriction or thrombotic signs as well as no retinal detachment. The oculomotor function remained intact. The blockade of ipsilateral ciliary ganglion parasympathetic fibers by mepivacaine may be the responsible mechanism. Systemic pathways as drug-drug interactions seem to be unlikely involved. Importantly, all three patients tolerated the same procedure previously or at a later date without any complication. Overall, our thoroughly elaborated risk management could not determine the causative factor explaining the observed ocular complications just in the current occasion and not at other time points.

**Conclusions:**

Doctors should be aware and patients should be informed about such rare complications after subconjunctival local anesthetics administration. Adequate risk management should insure patients’ safety.

## Background

Generally, local anesthetics are well tolerated, however, several serious adverse events, local or systemic, are reported. Overall, ocular complications are commonly rare and have been reported as transient vision loss or amaurosis [1–6], temporary paralysis of oculomotor muscles and cranial nerves III, IV and VI with diplopia [1, 6], mydriasis [1], Horner syndrome [1, 7], ptosis [8] and accommodation problems with both local ophthalmological and dental anesthesia. According to different authors and manifestation patterns, the prevalence after dental procedures is reported to be 1:1000 to 0,1% [1] or even 0,7% [9]. Usually, these complications are transient and disappear with ending of anesthetic effect. Several mechanisms and patterns as inadvertent intravascular injection or direct diffusion of local anesthetic solution to the eye socket after dental procedures as well as myotoxic effects are proposed [5, 6, 8]. By interaction with eye vegetative nervous system, local anesthetics could affect the nerve endings directly, promoting sympathetic or parasympathetic damaging patterns [1, 6]. Here, we present three cases with temporary amaurosis after subconjunctival application of mepivacaine 2% solution for laser cyclophotocoagulation (CPC) in patients with advanced refractory glaucoma.

## Case presentation

### Patient 1

**Fig. 1:**
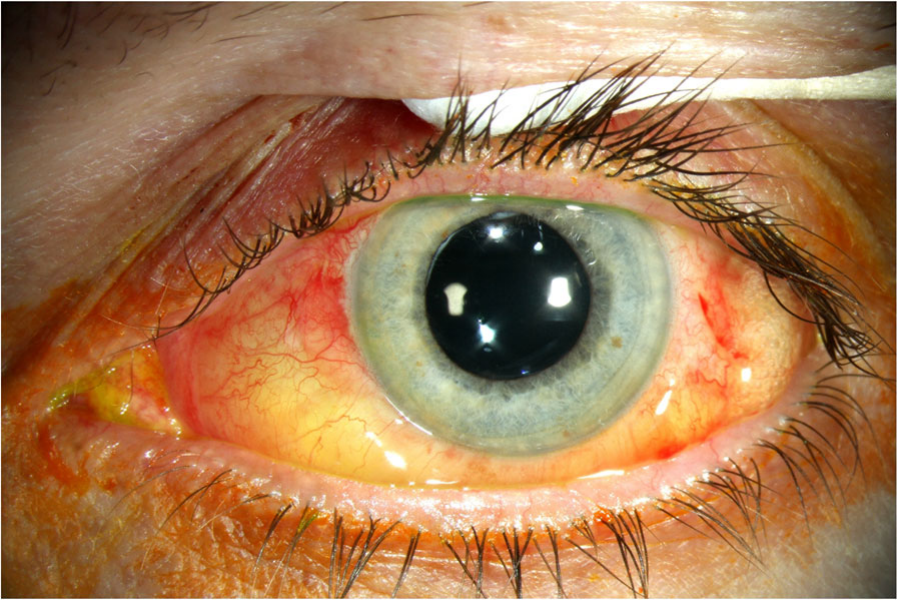
Anterior eye segment examination showing mydriasis soon after the local anesthetics injection (patient 1). The picture was taken after eye pressure measurement with fluorescein (yellow) dyeing

A 54-year-old man with 11-year advanced refractory glaucoma history was hospitalized in May 2017 for laser CPC on both eyes, as intraocular pressure (IOP) goal of ≤15 mmHg could not be reached by maximal tolerable topical antiglaucomatous medicine (Brinzolamid, Brimonidin, Bimatoprost, Timolol eye drops) after previous filtering glaucoma surgery three times on the left eye. The last visual acuity from March 2017 was 1.0 sc (decimal visual acuity) with proper light reaction on both eyes. On the left eye, there was an absolute scotoma over three quadrants stage Aulhorn IV-V. The optical coherence tomography (OCT) of the Optic Nerve head showed circularly reduced nerve fibers (BMO Rim Analysis). Funduscopic, sharp edged and pale Optic Nerve head with a cup disc ratio (CDR) of 1.0 were seen on both sides. The patient’s medical history comprised benign prostate hyperplasia without any medication use. The CPC on the right eye was performed the day before without complications. However, the CPC on his left eye on the following day caused a temporary amaurosis. The procedure was performed as follows: Topical anesthetic drops (procaine 0.5%) were given, followed by subconjunctival injection of 3–4 ml mepivacaine 2% solution (Scandicaine® 2%) using a BD Microlance 3 30G ½ needle distal of the limbus near the lower fornix. A good conjunctiva bladder was built, confirming correct subconjunctival injection. A few minutes later, the patient reported progressive vision deterioration, not experienced the day before. A visual acuity of no light perception, mydriasis (Fig. 1) without direct and consensual light response was observed. The CPC has not been performed. The IOP was 12 mmHg (appl.). Funduscopic, a normal optic disc, no signs of vascular occlusion or retinal detachment were seen. The OCT did not show any differences to the previous recordings. After about 90 min, the patient noticed a gradually increasing light perception with a visual acuity of 0.32 sc. Meanwhile, the urgently performed cranial MRI showed no abnormalities of the bulbus, the eye socket or the retrobulbar space. The visual acuity had risen to 1.0 sc about 20 h after local anesthetic application. No paresthesia or skin blanching around the affected left eye, no typical systemic side effects of local anesthetics or general condition impairment were observed. The oculomotor function was fully preserved.

### Patient 2

**Fig. 2:**
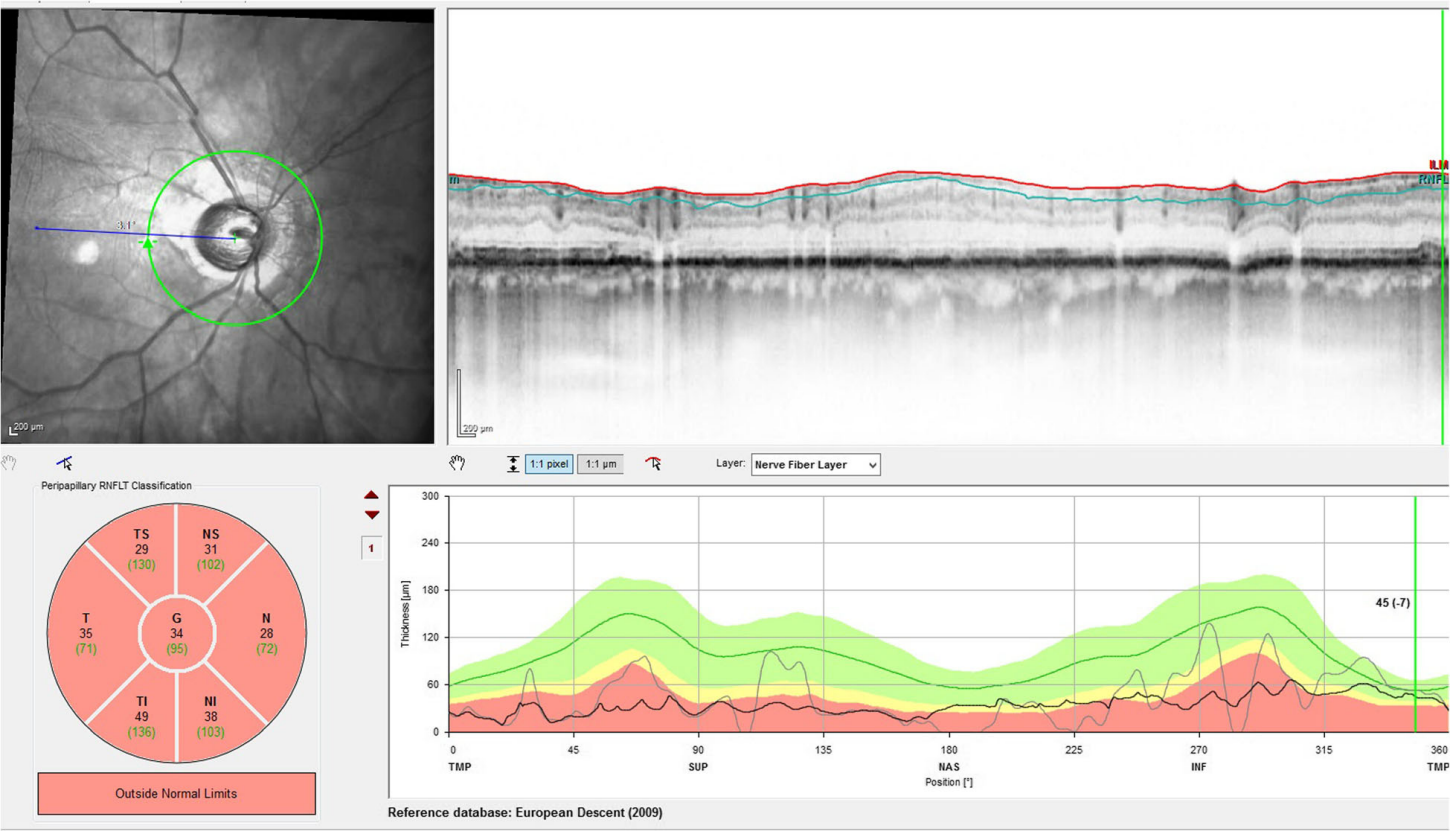
Optical coherence tomography of the papilla with circularly reduced nerve fiber, end stage glaucoma (patient 2)

**Fig. 3:**
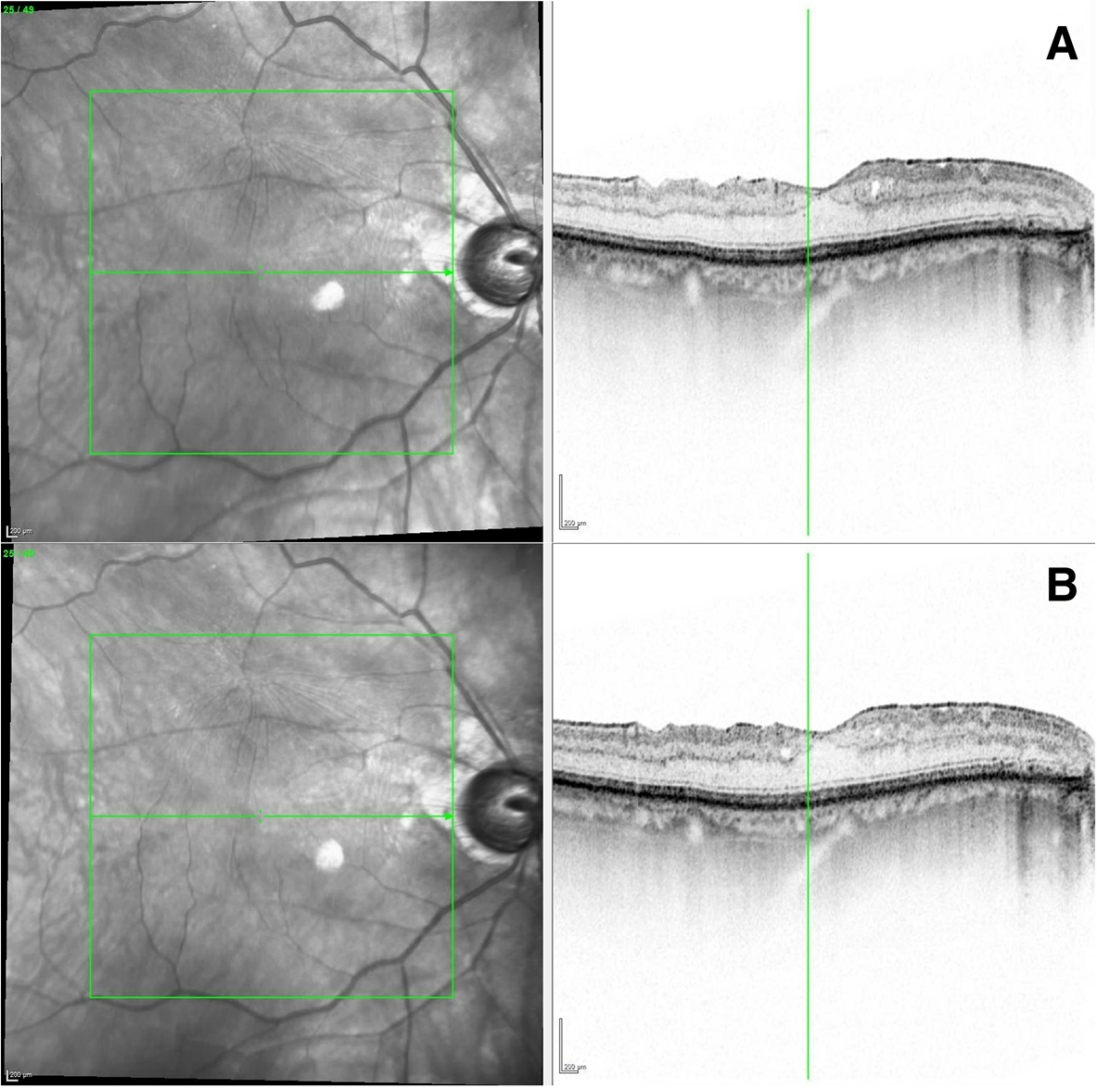
Optical coherence tomography of the macula before (**a**) and after (**b**) the subconjunctival injection. Intraretinal cysts and epiretinal gliosis remain stable (patient 2)

A 69-year-old man with oculus ultimus experienced almost identical complication course on the same day, treated only a few minutes later than our first patient. On the right side, chronic open-angle glaucoma was first diagnosed in 2014, with the highest measured IOP of 65 mmHg. The patient’s medical history was remarkable for slight mood depression and anxiety disorder associated with the history of leg amputation because of thrombosis and cerebral insult, atrial fibrillation, arterial hypertension, gout, thyroid dysfunction, and restricted renal function. The patient’s co-medication comprised pregabalin, rivaroxaban, amlodipine, ramipril, torasemide allopurinol, L-thyroxine, dipyrone, pantoprazole, and high-dose vitamin D. The last visual acuity was 0.32 sc (decimal visual acuity) with adequate light reaction. Optic Nerve damage was diagnosed previously with a circular absolute scotoma stage Aulhorn V under topical antiglaucomatous therapy (Dorzolamid and Clonidophtal, intolerance of any other substance). The OCT of the Optic Nerve head showed a circularly reduced nerve fiber (BMO Rim Analysis, Fig. 2). Funduscopic, sharp edged and pale Optic Nerve head with a CDR of 1.0 was described as glaucoma fere absolutum. In summary, he already showed a terminal glaucoma on his ultimate eye when first presented in our eye hospital. Hence, there was no other option than the first CPC in 2015 and re-performing it in 2017 because of insufficient pressure-reducing effect. The preparation and mepivacaine 2% injection were carried out in the same way and from the same ophthalmologist as for the first patient. After mepivacaine injection, the patient experienced vision loss to no light perception along with mydriasis and absence of direct and consensual light response. Thus, the CPC has been postponed. As in the first case, the ophthalmological examination, inclusive urgently performed cranial MRI, revealed no abnormalities or changes to prior findings (Fig. 3). In the next morning, the visual acuity of the right eye was fully recovered to 0.6 sc with an IOP at 14 mmHg (appl.). The oculomotor function was fully preserved. No other symptoms were detectable.

### Patient 3

The 64-year-old patient was treated about 9 months later than the aforementioned two patients in February 2018. Chronic open-angle glaucoma was first diagnosed in June 2012 on both eyes, with the highest measured IOP of 43 mmHg. The patient did undergo previous filtering glaucoma surgery three times without sufficient IOP lowering or stable visual fields. That is the reason why CPC has been performed twice in March and May 2017 but without complications. The patient’s medical history comprised arterial hypertension treated with ramipril. At the emergency presentation, the visual acuity was measured as 0.1 sc (decimal visual acuity) with a decompensation of the IOP at 44 mmHg even with full antiglaucomatous medication (oral acetazolamide 3 × 250 mg daily and Clonidophtal, Tafluprost, Dorzolamid and Timolol eye drops). Optic Nerve damage was diagnosed previously with an absolute scotoma stage Aulhorn V with participation of the blind spot. The OCT of the Optic Nerve head showed a temporal inferior reduced nerve fiber (BMO Rim Analysis). Funduscopic, a sharp edged Optic Nerve head with a CDR of 0.9–1.0 was documented. After lowering the IOP locally and systemically, preparation and mepivacaine 2% injection were performed in the same way, while from another ophthalmologist. A visual acuity of no light perception along with mydriasis with restricted pupil motility was observed. As in the first two cases, no acute abnormalities were seen in the ophthalmological examination. The visual acuity slowly recovered to 0.7 sc with an all-time stable IOP within 24 h. The oculomotor function was fully preserved. No further symptoms were detected.

## Discussion and conclusions

We described three cases with temporary amaurosis after subconjunctival mepivacaine 2% administration for laser CPC in patients with advanced refractory glaucoma, two of which have been experienced on the same day and the third one about 9 months later. In our ophthalmological clinic, 1031 CPC procedures (using local anesthetics in nearly 95% of cases) were carried out in the past 3 years, without such complications experienced before. These three cases are not only special from a medical point of view as they represent complications that are little known so far, but also from the point of view of adequate risk management. The risk manager of our medical school was involved and elaborated an investigation plan.

For convenience, the relevant questions necessary to be addressed were separated into patient-related, drug-related and administration technique-related. *Patient-related aspects were*: Which patients’ similarities predisposed to the observed complication? Why were at least two of them affected exactly on the same day? *Drug-related peculiarities:* May any pharmaceutical problem, e.g. incorrect substance or vasoconstrictor co-mixture (e.g. adrenaline) in the local anesthetics vials be responsible? Could any drug-drug interaction explain the observed side effects? *Administration technique-related:* Could any technical, procedural problem during local anesthetic administration be the clue? Further questions were as well: Which mechanism underlies the affection pattern? Would the re-exposition lead to the same pathological reaction? How could such complications be anticipated as well as be prevented in future? The patients’ thorough medical history analysis failed to reveal any peculiarities associated with general condition or co-morbidities. Moreover, in our first patient, the same procedure a day before performed on his right eye by the same ophthalmologist was well tolerated. Remarkably, the re-exposition with mepivacaine in the frame of laser CPC about 1,5 month later proceeded without any complication in both patients (the other, right eye of the first patient and the same eye of the second patient were treated). According to literature, the majority of patients experienced ocular side effects only once despite of repeated local anesthetics exposures [1]. However, one case report described diplopia and external rectus muscle palsy in a woman on three consecutive local anesthesia with mepivacaine in dental settings [10]. Most cases with ocular complications are reported for lidocaine (57%), articaine (19,3%), procaine (10,5%), and mepivacaine (7,9%) [5]. In the vast majority of cases (94,5%), anesthetic solutions contained vasoconstrictors (e.g. adrenaline) [5]. The prompted chemical analysis of the used mepivacaine vials via an independent laboratory (the Central Laboratory of German Pharmacists, Eschborn, Germany) excluded accidental adrenaline contamination during manufacturing process, and the investigated solutions contained solely the declared mepivacaine. Mepivacaine is a local anesthetic of the amide type with rapid action onset and reversible blockade of vegetative, sensor und motor nerve fibers as well as the heart conduction system. Our clinical pharmacological medication analysis revealed no drug-drug interactions between mepivacaine and patients’ co-medication. Moreover, the event time course (appearance after few minutes after mepivacaine administration), affection of only ipsilateral eye, medical history with no previous complications to mepivacaine as well as well-tolerated re-exposition argues against a causative role of drug-drug interactions and suggests a local affection as the cause. Both ophthalmologists were well experienced in the procedure and performed it without any remarkable technical difficulty which was confirmed by co-assisting colleagues as well.

Local anesthetics can elicit ocular complications during dental procedures reaching eye socket via vascular, neurological and lymphatic pathways [11, 12]. Individual regional vascular-anatomical variations (anomalies) could predispose to such side effects [6]. In case of ocular local anesthesia, the affection way may be shorter and easier. Several affection mechanisms are suggested in the frame of dental local anesthesia. An inadvertent intra-venous anesthetic injection could lead to cavernous sinus syndrome with preferential affection of nearest three oculomotor nerves (III, IV and VI) causing diplopia and muscle palsy. An accidental intra-arterial anesthetic injection may result in arterial terminal branch constriction either directly by admixtured vasoconstrictor or via sympathetic reflex activation due to direct mechanical arterial wall trauma resulting in transient pain, vision loss due to retinal vasoconstriction, regional skin and mucosal pallor as well as sensory deficits [5, 6]. Neither of abovementioned symptoms was seen in our patients. Direct anesthetic tissue diffusion towards eye socket along with diffusion via bony openings is further pathways during dental anesthesia [1, 6, 12]. Systemic symptoms (e.g. vasovagal reactions, tachyarrhythmia, palpitation, and anaphylactic reactions) are reported as well, however, have not been seen in our patients. In some cases, an ocular muscles mechanical trauma as well as myotoxic and neurotoxic action of anesthetic solutions are proposed as possible mechanisms [8, 13].

Generally, two affection patterns, sympathetic and parasympathetic, have been discussed [1, 6]. Preexisting Optic Nerve damage may increase the vulnerability to respective side effects. The parasympathetic affection pattern, resulting from blockage of ocular parasympathetic neural fibers at the ciliary ganglion level, located between Optic Nerve and external rectus muscle of the eye (eye socket) and facilitating pupil (ciliary muscle) constriction, would manifest as mydriasis, accommodation loss and absent ipsilateral direct and consensual light reflexes [6]. Thus, we proposed that this may be the responsible mechanism also in our patients. It could be speculated that the anesthetic solution reached the ciliary ganglion via ciliary vessels exerting a neurotoxic reaction or ganglion anesthesia. Overall, despite of our thorough search, we could not find a satisfying answer to the justifiable question, why exactly these three patients from about annually treated 300 were affected.

We speculate that mepivacaine subconjunctival application provoked ipsilateral temporary amaurosis, mydriasis and light reflex absence by neurotoxic action on parasympathetic fibers at the ciliary ganglion level. Doctors should be aware and patients should be informed about such rare complications after local anesthetics administration. Adequate risk management should insure patients’ safety.

## Data Availability

The datasets used and/or analyzed during the current study are available from the corresponding author on reasonable request.

## Abbreviations

CDR: Cup disc ratio
CPC: Cyclophotocoagulation
IOP: Intraocular pressure
MRI: Magnetic resonance imaging
OCT: Optical coherence tomography
sc: Sine correctione
